# Long-Term Effectiveness and Safety of Tofacitinib in a Nationwide Veterans Affairs Cohort of Ulcerative Colitis Patients

**DOI:** 10.1093/crocol/otaf037

**Published:** 2025-07-09

**Authors:** Nabeel Khan, Ramaswamy Sundararajan, Nadim Mahmud

**Affiliations:** Department of Gastroenterology, Corporal Michael J Crescenz VA Medical Center, Philadelphia, PA, USA; Division of Gastroenterology, University of Pennsylvania, Perelman School of Medicine, Philadelphia, PA, USA; Department of Gastroenterology, Corporal Michael J Crescenz VA Medical Center, Philadelphia, PA, USA; Department of Gastroenterology, Corporal Michael J Crescenz VA Medical Center, Philadelphia, PA, USA; Division of Gastroenterology, University of Pennsylvania, Perelman School of Medicine, Philadelphia, PA, USA

**Keywords:** ulcerative colitis, tofacitinib, biologics, effectiveness, safety

## Abstract

**Background:**

There is limited real-world data on the long-term effectiveness and safety of tofacitinib among ulcerative colitis (UC) patients, especially among the elderly. Our aim was to evaluate these parameters among UC patients who had remained on the drug 1 year after initiation of therapy.

**Methods:**

We conducted a retrospective cohort study, utilizing the US National Veterans Affairs Healthcare System, including patients with UC who received tofacitinib. The primary endpoint was effectiveness at the end of follow-up. The secondary endpoint was to evaluate if the effectiveness was different among the elderly compared to the young. Adverse events associated with the drug, like herpes zoster, major adverse cardiovascular events, deep vein thrombosis, as well as infections and malignancy during follow-up were also assessed.

**Results:**

In total, 159 patients were included in the study, among whom 51 were in the elderly group and 108 were in the younger group. The median duration of follow-up was 1.47 years (range, 0.01-5.49 years). Effectiveness of tofacitinib among the cohort was 56.60% (90 out of 159 patients). The effectiveness was higher in the elderly (*P* = .005).

**Conclusion:**

In this nationwide cohort of UC patients, the effectiveness of tofacitinib was seen in a little over half the number of patients, with higher rates of effectiveness reported among the elderly. No new safety concerns were raised, especially among the elderly.

## Introduction

Ulcerative colitis (UC) is a chronic inflammatory disorder characterized by increased frequency of bowel movements, bleeding, and urgency.^1^ It is primarily managed by different formulations of mesalamine with some patients requiring more potent immunosuppressive therapy.^2^ The first of these to be approved were antagonists to tumor necrosis factor (anti-TNF) followed by those to α4β7 integrin.^3–5^ However, a sizable proportion of patients either do not respond to these agents, or over time fail to maintain a sustained response. To treat these patients, tofacitinib an oral, small molecule that inhibits all Janus Kinase (JAKs) and preferentially inhibits JAK1 and JAK3, was approved for the treatment of UC.^6,7^

The efficacy and safety of tofacitinib were evaluated in the two 8-week OCTAVE induction studies and a 52-week maintenance study^7^ as well as in OCTAVE OPEN, a long-term extension study where the efficacy among different groups was evaluated up to 36 months.^8^ While clinical trials are required for regulatory approval, they are not reflective of real-world data as studies have shown that only a quarter of patients would be eligible to participate in such clinical trials.^9^ Furthermore, elderly patients are often excluded from trials leading to paucity of data in that age group. While there are real-world studies that have evaluated the efficacy and safety of tofacitinib these have mostly followed patients for a limited period.^10–15^ Hence, there is lack of data on the long-term effectiveness of the drug in the real-world setting.

Our aim was to evaluate the long-term effectiveness and safety of tofacitinib among UC patients who had remained on the drug 1 year after initiation of therapy as most of the patients who stop the drug do so within the first year and studies have evaluated the effectiveness of tofacitinib at the end of 52 weeks.^16^ To achieve this objective, we identified patients treated with tofacitinib in a nationwide cohort of UC patients followed in the Veterans Health Administration. Our secondary aim was to evaluate if the clinical course was different in the elderly compared to the younger population.

## Materials and Methods

### Cohort Characteristics

This was a retrospective study utilizing a well-established Veterans Affairs Healthcare System (VAHS) cohort of patients with UC. This included patients who were followed in the VA and had a diagnosis of UC. Briefly, our source cohort was created using a previously validated algorithm: (1) ICD-9/ICD-10 diagnosis code for UC; (2) ≥ 1 outpatient encounter in the VA; (3) at least 1 outpatient pharmacy claim for any UC medication—(i) Mesalamine, (ii) thiopurines, (iii) antitumor necrosis factor (TNF) agents, (iv) combination of thiopurines and anti-TNF agents, (v) vedolizumab, and (vi) tofacitinib; and (4) at least 2 prescriptions of 1 distinct medication in the above 6 UC medication groups.**^17^**

Our inclusion criteria were: (1) patients who were initiated on tofacitinib therapy for UC in the VAHS and (2) remained on tofacitinib for at least 1 year after initiation of therapy. Demographics, baseline endoscopic evaluation, and baseline laboratory values of interest were obtained for each patient. The study cohort was divided based on the age at the time of initiating tofacitinib therapy into the elderly group who were 65 years and older and the younger group who were less than 65 years old. To ascertain outcomes, follow-up began 1 year after tofacitinib initiation, and patients were censored at the first occurrence of: (1) colectomy, (2) death, or (3) stopped taking the drug.

### Outcomes

The primary endpoint was the effectiveness of tofacitinib therapy at the end of follow-up. This was a composite outcome that included patients who met the following criteria: (1) remaining on tofacitinib at the end of follow-up, (2) not requiring steroids at any point, 12 months after initiation, and (3) no hospital admission for UC or change in UC medications at any point, 12 months after initiation.^16^ Secondary outcomes included mucosal healing which was defined as a reduction in Mayo endoscopic sub score from 2/3 to 1/0 or from 1 to 0.^18^ This was limited to patients who had a baseline endoscopic evaluation as well as an endoscopy, 1 year following tofacitinib initiation. All study data were collected through individual chart review. We also evaluated reasons for stopping tofacitinib. We also assessed side effects that are thought to be associated with tofacitinib such as herpes zoster (HZ), major adverse cardiovascular events (MACE) and venous thromboembolism. Furthermore, we identified whether patients had developed any infection or malignancy during follow-up. Infections were classified as those treated in the outpatient setting or those that required hospitalization.

### Statistical Analysis

Descriptive statistics were computed as means and standard deviations for normally distributed continuous variables and as medians and interquartile ranges for skewed variables. Frequencies and percentages were reported for categorical variables. Group comparisons were evaluated using *t*-tests, Wilcoxon rank-sum tests, or chi-square tests, as indicated. We additionally performed a multivariable Cox regression analysis where the outcome was framed as time to ineffective treatment. Forward stepwise selection was used to identify variables significantly associated with this outcome, with a primary exposure of age category (younger vs. elderly). Hazard ratios, 95% confidence intervals (CIs), and adjusted survival curves were presented.

### Ethical Considerations

This project received Institutional Review Board approval from the affiliated medical center.

## Results

There were 159 eligible patients, with 51 in the elderly category (median age 71 years [interquartile range {IQR} —69, 73]) and 108 young patients (median age 42.5 years [IQR—36, 52]). Table 1 presents the baseline characteristics of these 159 patients distributed among the elderly and young. Overall, 45.27% had left sided colitis at baseline prior to initiation of tofacitinib (51.02% in the elderly, 42.42% in the young).

**Table 1.** Baseline characteristics.

Characteristic	Elderly (*n* = 51)	Young (*n* = 108)	*P* value
Age, yr, median, (range)	71 (69, 73)	42.5 (36, 52)	<.001
Male sex, *n* (%)	50 (98.04)	98 (90.74)	.04
Race, *n* (%)			
White	41 (80.39)	79 (73.15)	.42
African American	6 (11.76)	13 (12.04)	
Decline to answer	2 (3.92)	9 (8.33)	
Asian	0 (0.00)	1 (0.93)	
American Indian or Alaskan Native	1 (1.96)	3 (2.78)	
Hispanic	1 (1.96)	3 (2.78)	
	(***n* = 49)**	**(*n* = 99)**	
Baseline endoscopy—extent, *n* (%)‼≈			
0	0 (0.00)	1 (1.01)	.95
1	8 (16.33)	13 (13.13)	
2	25 (51.02)	42 (42.42)	
3	16 (32.65)	43 (43.43)	
Baseline endoscopy—severity, *n* (%)			
0	0 (0.00)	1 (1.01)	.23
1	15 (30.61)	25 (25.25)	
2	11 (22.45)	36 (36.36)	
3	23 (46.94)	37 (37.37)	
Disease duration, yr, median (IQR)	6.01 (2.71, 9.77)	5.45 (2.44, 12.64)	.95
Albumin, median (IQR)^†^	3.75 (3.6, 4)	4 (3.7, 4.3)	.02
Hemoglobin, median (IQR)^‡^	13.1 (11.4, 13.6)	13.9 (12.4, 15)	.21
White Blood Cell count, median (IQR)*•*	9.1 (7.3, 12.5)	7.5 (5.7, 9.99)	.03
Platelet count, median (IQR)^‖^	286 (244, 346)	286 (242, 341)	.64
Body mass index, median (IQR)	29 (26, 32)	28 (24, 33)	.48
Smoking, *n* (%)			
Never smoker	29 (56.86)	82 (75.93)	.004
Former smoker	19 (37.25)	18 (16.67)	
Current smoker	3 (5.88)	8 (7.41)	
Prior exposure, *n* (%)			
1 anti-TNF	11 (21.57)	35 (32.41)	.059
More than 1 anti-TNF	2 (3.92)	16 (14.81)	
Anti-TNF and vedolizumab and/or Ustekinumab	26 (50.98)	33 (30.56)	
Vedolizumab or Ustekinumab only	1 (1.96)	3 (2.78)	
No exposure	11 (21.57)	21 (19.44)	
Concomitant exposure			
5-ASA	13 (25.49)	26 (24.07)	.85
Immunomodulators—Thiopurines/Methotrexate	0 (0.00)	0 (0.00)	NA
Dose			
5 mg twice daily	35 (68.63)	62 (57.41)	.18
10 mg twice daily	16 (31.37)	46 (42.59)	

Data are presented as median (IQR) for continuous measures, and *n* (%) for categorical measures.

‼Baseline endoscopy: 2 out of 51 in the elderly and 9 out of 108 in the young did not have a baseline endoscopy recorded.

≈Extent was evaluated using Montreal classification consisting of E1—ulcerative proctitis, E2—left sided UC (distal UC), E3—Extensive UC. Since 1 patient in the younger group had no inflammation in their colonoscopy report at baseline, they were marked as having 0 for extent to signify no inflammation and normal colonoscopy.

^†^Albumin: 15 out of 51 in the elderly and 18 out of 108 in the young did not have albumin levels measured at baseline.

^‡^Hemoglobin: 5 out of 51 in the elderly and 11 out of 108 in the young did not have hemoglobin levels measured at baseline.

•White Blood cell count: 5 out of 51 in the elderly and 11 out of 108 in the young did not have white blood cell counts measured at baseline.

^‖^Platelet count: 5 out of 51 in the elderly and 11 out of 108 in the young did not have platelet counts measured at baseline.

**Figure 1.**
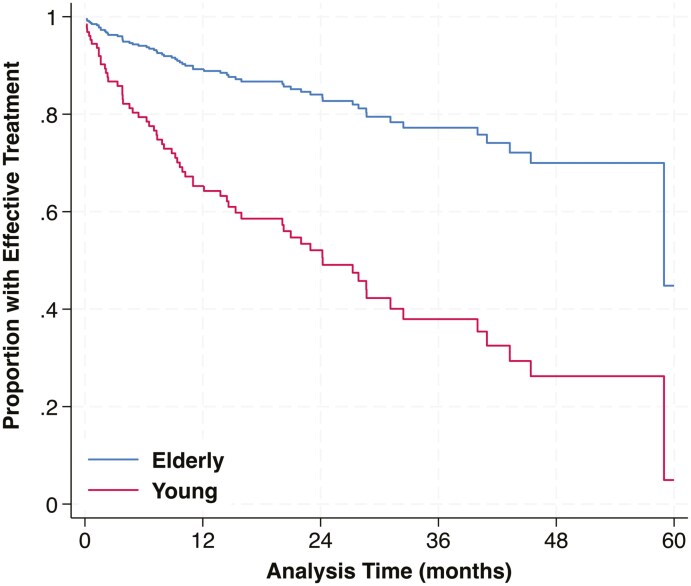
Proportion of patients with tofacitinib effectiveness in months, stratified by age.

The median duration of follow-up was 1.47 years (range, 0.01-5.49 years). Over the course of follow-up, 108/159 (67.92%) remained on tofacitinib at the end of follow-up, with effectiveness being 56.60% (90 out of 159 patients). Among these, 82.35% (42/51) in the elderly remained on the drug with effectiveness observed in 72.55% of patients (37/51). 61.11% (66/108) of the young remained on the drug with effectiveness seen in 49.07% (53/108) patients. We observed the effectiveness of tofacitinib to be higher among the elderly. Mucosal healing in the last endoscopic evaluation after drug initiation, overall, was 56.84% (54/95) (68.75% in the elderly, 50.79% in the young) (Table 2). In our multivariable Cox regression model with an outcome of time to ineffectiveness of treatment, among all available covariates, we noted that younger patients, prior use of a single anti-TNF, or of an anti-TNF and vedolizumab/ustekinumab were significantly associated with a higher hazard of ineffective treatment (Table 3). Figure 1 depicts a Kaplan Meier curve which showed a steeper decline in the proportion of patients in the younger group compared to the elderly group, thus indicating a greater risk for ineffective treatment with tofacitinib in the younger group over time. Among patients on a 10 mg twice daily baseline dose, 56.25% (9/16) in the elderly and 34.78% (16/46) in the young, had their dose reduced to 5 mg twice daily. Escalation of dose from 5 mg twice daily at baseline to 10 mg twice daily was observed in 2.86% (1/35) in the elderly and 22.58% (14/62) in the young.

**Table 2.** Effectiveness and mucosal healing.

	Elderly (*n* = 51)	Young (*n* = 108)	*P* value
Effectiveness, *n* (%)	37 (72.55)	53 (49.07)	.005

Mucosal healing (Mayo 2/3 to 1/0 or 1 to 0)	Elderly (*n* = 32)	Young (*n* = 63)	*P* value
	22 (68.75%)	32 (50.79%)	.09

**Table 3.** Multivariable cox regression model for time to ineffectiveness.

Covariates	Hazard Ratio	95% CI	*P* value
Young age (vs. Elderly)	3.75	(1.94, 7.24)	<.001
Prior exposure to 1 anti-TNF (vs. not)	2.96	(1.54, 5.68)	.001
Prior exposure to 1 anti-TNF and vedolizumab/Ustekinumab (vs. not)	2.30	(1.15, 4.61)	.019

Among this cohort, 51/159 (32.08%) patients discontinued tofacitinib. The most common reason for discontinuation of the drug in both groups was loss of response. This was seen in 5.88% of the elderly and 26.85% of the young. Two patients in the younger group stopped the drug after they achieved clinical remission (Table 4).

**Table 4.** Reason for discontinuation.

Reason for discontinuation	Elderly (*n* = 51)	Young (*N* = 108)
Loss of response	3 (5.88)	29 (26.85)
Adverse event	3 (5.88)	2 (1.85)
Colectomy—Adenocarcinoma colon	2 (3.92)	1 (0.93)
Colectomy—Disease exacerbation	0 (0.00)	2 (1.85)
Infection	0 (0.00)	1 (0.93)
Other Malignancy	1 (1.96)	0 (0.00)
Clinical remission	0 (0.00)	2 (1.85)
Cardiac risk factors	0 (0.00)	1 (0.93)
Patient factors (rheumatology, compliance)	0 (0.00)	4 (3.70)

Infections treated in the outpatient had an incidence rate (IR) of 8.86 per 100 patient-years (PY); (95% CI, 5.56-13.43), followed by infections requiring hospitalization, with an IR of 3.34 per 100 PY; (95% CI, 1.53-6.35). The incidence of non-melanoma skin cancer (NMSC) was 1.81 per 100 PY (95% CI, 0.59-4.21). The incidence of HZ was IR—0.71 per 100 PY; (95% CI, 0.09-2.57) and all cases were noted among patients who were not immunized. Other adverse events of interest are summarized in Table 5.

**Table 5.** Safety.

Safety outcomes	Elderly (*n* = 51)	Young (*n* = 108)	Overall (*n* = 159)
	IR (95% CI)	IR (95% CI)	IR (95% CI)
Infections treated in OP setting	10.91 (5.44-19.49)	7.46 (3.71-13.30)	8.86 (5.56-13.43)
Infections requiring hospitalization	2.54 (0.52-7.43)	3.97 (1.46-8.65)	3.34 (1.53-6.35)
Malignancy excluding NMSC	3.33 (0.91-8.54)	2.52 (0.69-6.44)	2.87 (1.24-5.65)
NMSC	1.69 (0.21-6.12)	1.89 (0.39-5.55)	1.81 (0.59-4.21)
Adverse events			
MACE	0.84 (0.02-4.68)	0	0.36 (0.01-1.99)
Herpes Zoster	0.83 (0.02-4.60)	0.62 (0.02-3.46)	0.71 (0.09-2.57)
DVT	0.83 (0.02-4.64)	0	0.36 (0.01-1.98)

Abbreviations: IR, incidence rate per 100 patient—years; OP, Outpatient; CI, confidence interval; NMSC, non-melanoma skin cancer.

## Discussion

In a US-based nationwide cohort of UC patients on tofacitinib with a median follow-up of 77 weeks (1.47 years), effectiveness of the drug was seen in a little over half of the total cohort. Effectiveness reported in the elderly was significantly higher than the younger group. Two-thirds of the patients who had remained on the drug after the completion of 1 year continued the drug.

There is limited real-world data on long-term effectiveness and safety of tofacitinib, and to the best of our knowledge, there is no study that has evaluated these parameters after 1 year of remaining on the drug. This is an important aspect as most patients who stop the drug, do so within the first year. Furthermore, these studies had limited number of patients and variable periods of follow-up. A UK based cohort study had a median follow-up of 53 weeks, and only 27 patients had completed 104 weeks of follow-up.^19^ A US-based retrospective cohort study, had 60 patients who were evaluated for 78 weeks.^20^ Chaparro et al., who defined clinical remission as a partial Mayo score ≤ 2, reported 49% (20 out of 41) patients in remission at the end of 36 months of follow-up, where median duration of follow-up was 18 months.^21^ A Dutch-based cohort study utilizing a registry, defined clinical remission as Simple Clinical Colitis Activity Index ≤ 2 and reported a combined outcome of corticosteroid (CS)-free clinical remission of 31.8% (34 out of 107) at 104 weeks of follow-up.^22^

Our study has a longer duration of follow-up than the other real-world studies, having a median follow-up of 77 weeks (Maximum follow-up—286 weeks [5.5 years]) after the completion of 1 year on therapy. While 68% of the patients remained on the drug, CS-free effectiveness was seen in 56.60% of patients overall. It was significantly higher in the elderly group (72.55% vs. 49.07%, *P* = .005). The main reason for this difference was the loss of response as 26.85% (29 out of 108) in the younger group versus 5.88% (3 out of 51) in the older group discontinued the drug due to loss of response. These findings have been previously observed in our short-term data.^16^ Additionally, our multivariable cox regression model and survival analysis curve revealed that patients in the younger group had a higher risk for ineffective treatment with tofacitinib. While we cannot state with certainty the reason behind this observation, there are immunological differences between the young and the elderly. As aging is associated with both B and T cell Immunosenescence as well as alterations in mucosal immunity, it is plausible that the immune system remodeling characteristics of the elderly may influence drug response.^23^ Additionally, mucosal healing while not statistically significant, possibly due to the smaller number of patients who underwent endoscopic evaluation, was higher in the elderly and almost two-thirds of this group achieved mucosal healing. In the multivariable analysis, the only other factor predicting effectiveness was prior use of a single anti-TNF, or of an anti-TNF and vedolizumab/ustekinumab which were significantly associated with a higher hazard of ineffective treatment.

There was a total of 9 infections, and no pattern was observed. Coronavirus disease 2019 (COVID-19) was the most common, affecting 2 of the 3 elderly patients and 1 of the 6 younger patients while the remainder affected different sites with no cases of bacterial pneumonia. All the cases of HZ took place in the non-vaccinated group further highlighting the importance of vaccination in this population. There were cases of NMSC in both the elderly and the young and although further studies are needed, it may be prudent that patients on tofacitinib should undergo an annual skin exam. Tofacitinib has been associated with thromboembolic disease and MACE, both of which are required as boxed warnings.^24^ The incidence of deep vein thrombosis (DVT) in our cohort was low (IR, 0.36 per 100 PY; 95% CI, 0.01-1.98) and resembles that previously reported (0.06-0.46).^21,25^ The patient with DVT had other risk factors like old age, obesity and was on prednisone.^26^ Incidence of MACE in prior literature was 0.27 per 100 PY which resembled our reported incidence of 0.36 per 100 PY.^25^ The patient who developed MI, was old, obese and had a history of hypertension.**^27^** Our study highlights that care may need to be taken among patients with preexisting risk factors.

A major strength of our study is the utilization of this nationwide cohort from the VAHS, which serves over 9 million veterans every year.**^28^** Every chart was individually reviewed to accurately ascertain all the findings. As these patients were receiving medications in the VA and were being followed closely, we feel we were able to accurately capture the safety outcomes. However, our study is not without its limitations. Our cohort consists of predominantly male and older patients, limiting the external validity of the study. There are inherent limitations due to its retrospective nature. We were precluded from using biomarkers such as erythrocyte sedimentation rate, C-reactive protein , and calprotectin since they were infrequently ordered. While no new safety signals were observed, these findings are limited by the relatively small number of patient years of follow-up.

## Conclusion

In this US-based nationwide cohort of UC patients, tofacitinib achieved effectiveness in a little over half of the overall cohort of patients, with higher rates of effectiveness identified among the elderly. No new safety concerns were raised. These findings reinforce the role of tofacitinib as an effective and safe long-term therapy for the management of UC among all age groups.

## Data Availability

The data for this manuscript cannot be made available in accordance with the HIPAA rules. However, de-identified data (without patient name and SSN), can be made available upon reasonable request.
